# Advancing Microcirculation Research in Advanced Heart Failure: The Case for Methodologic Consensus

**DOI:** 10.1016/j.jacadv.2026.103148

**Published:** 2026-08-15

**Authors:** John C. Greenwood, Joyce W. Wald, James W. Schurr

**Affiliations:** a Department of Emergency Medicine, Center for Resuscitation Science, Perelman School of Medicine at the University of Pennsylvania, Philadelphia, Pennsylvania, USA; b Department of Anesthesiology and Critical Care, Perelman School of Medicine at the University of Pennsylvania, Philadelphia, Pennsylvania, USA; c Division of Cardiovascular Medicine, Department of Medicine, Perelman School of Medicine at the University of Pennsylvania, Philadelphia, Pennsylvania, USA

We commend Baranowska et al^1^ for their study characterizing sublingual microcirculation across patients with chronic heart failure, resuscitated cardiogenic shock, recent left ventricular assist device implantation, and heart transplantation. Recruiting patients across the advanced heart failure spectrum is difficult and understudied. The perfused boundary region (PBR) is an inverse measure of endothelial glycocalyx thickness and the variable for which the GlycoCheck system was designed.^2^ Their finding of an elevated PBR suggesting persistent glycocalyx impairment despite hemodynamic resuscitation adds meaningful data in a population where microcirculatory biology remains poorly characterized.

However, the principal findings are difficult to interpret within the broader microcirculation literature based on the chosen methods. The authors departed from the 2018 European Society of Intensive Care Medicine consensus,^3^ which requires validated analysis methods and defines a variable set that includes total vessel density (TVD), perfused vessel density, proportion of perfused vessels, microvascular flow index, and heterogeneity index, which exists specifically to enable cross-study comparison. Baranowska et al^1^ report GlycoCheck-derived variables, omitting perfused vessel density and flow heterogeneity, the pathophysiologic hallmarks of microcirculatory dysfunction in shock. Healthy reference range values using the same GlycoCheck platform have reported a median microvascular density of 411 μm/mm^2^ and PBR median of 1.89 μm in healthy adults,^4^ values that align with Baranowska et al's PBR (2.2 μm) but differ substantially in TVD (851 μm/mm^2^). This pattern of consistency for PBR but variability across vessel density suggests the automated software performs reliably for glycocalyx assessment but poorly for microcirculation measurements. GlycoCheck-derived vessel density values are also an order of magnitude below consensus-derived measurements. Our group recently published a reference data set from ambulatory patients with chronic cardiovascular disease with a TVD of 24.5 ± 4.8 mm/mm^2^ using consensus methods.^5^

Acquisition methods also deviated from consensus protocols. Imaging durations of 10 to 90 minutes far exceed those of comparable studies and would be difficult for critically ill patients to tolerate. Acquiring 400 recordings before accepting a video for analysis increases the risk of selection bias, and Baranowska et al^1^ did not report discard criteria or imaging quality metrics, raising the possibility that algorithmic rejection of videos with intermittent or heterogeneous flow that may have biased the data set toward expected results.

Finally, the clinical state of the imaged cohorts further constrains interpretation. Measurements were obtained in stable patients on durable mechanical circulatory support, post-transplant recipients, and cardiogenic shock patients after initial resuscitation, with an unspecified proportion in active shock vs stabilized on support. Acute and chronic microcirculatory derangements reflect distinct biology and pooling them obscures important differences.

These concerns do not diminish the importance of the research question but limit the conclusions that can be drawn. Microcirculatory assessment holds genuine promise to elucidate mechanisms of organ injury in cardiogenic shock, and Baranowska et al^1^ have provided valuable information about vascular endothelial status in this population. Future studies grounded in consensus-aligned analysis, with comorbidity-matched controls and within-cohort stratification by acuity, support modality, and shock severity, will allow the field to build a comparable evidence base across centers and translate microcirculatory findings into personalized and actionable care.
